# The VPAC1 receptor: structure and function of a class B GPCR prototype

**DOI:** 10.3389/fendo.2012.00139

**Published:** 2012-11-16

**Authors:** A. Couvineau, E. Ceraudo, Y.-V. Tan, P. Nicole, M. Laburthe

**Affiliations:** Faculté de Médecine Xavier Bichat, INSERM 773/Centre de Recherche Biomédicale Bichat Beaujon (CRB3), Université Paris 7Paris Cedex 18, France

**Keywords:** GPCR, photolabeling, VPAC1, VIP, mutagenesis, inflammation, neuroprotection, molecular modeling

## Abstract

The class B G protein-coupled receptors (GPCRs) represents a small sub-family encompassing 15 members, and are very promising targets for the development of drugs to treat many diseases such as chronic inflammation, neurodegeneration, diabetes, stress, and osteoporosis. The VPAC1 receptor which is an archetype of the class B GPCRs binds Vasoactive Intestinal Peptide (VIP), a neuropeptide widely distributed in central and peripheral nervous system modulating many physiological processes including regulation of exocrine secretions, hormone release, foetal development, immune response … VIP appears to exert beneficial effect in neurodegenerative and inflammatory diseases. This article reviews the current knowledge regarding the structure and molecular pharmacology of VPAC1 receptors. Over the past decade, structure–function relationship studies have demonstrated that the N-terminal ectodomain (N-ted) of VPAC1 plays a pivotal role in VIP recognition. The use of different approaches such as directed mutagenesis, photoaffinity labeling, Nuclear Magnetic Resonance (NMR), molecular modeling, and molecular dynamic simulation has led to demonstrate that: (1) the central and C-terminal part of the VIP molecule interacts with the N-ted of VPAC1 receptor which is itself structured as a « Sushi » domain; (2) the N-terminal end of the VIP molecule interacts with the first transmembrane domain of the receptor where three residues (K^143^, T^144^, and T^147^) play an important role in VPAC1 interaction with the first histidine residue of VIP.

## Introduction

Vasoactive Intestinal Peptide (VIP) discovered by Said and Mutt (1970) is an ubiquitous 28-aminoacid neuropeptide that is widely distributed in central and peripheral nervous system. During the past 10 years, VIP was also identified in the immune system where it plays the role of a “cytokine-like peptide” (Delgado et al., 2004). VIP plays an important role in human physiology (Table 1) such as in development, growth, immune responses, circadian rhythms, neuronal and endocrine control, neuroprotective actions, and in the functions of the digestive, respiratory, reproductive, and cardiovascular systems (Laburthe et al., 2007). Associated to its large distribution and biological functions, VIP may also play a role in various pathologies (Table 1). It has been identified as a very promising agent in the treatment of inflammatory and neurodegenerative diseases (Gozes et al., 2003; Delgado et al., 2004). Indeed, VIP appears to be a very potent anti-inflammatory peptide in animal models of Crohn disease (Abad et al., 2003), rheumatoid polyarthritis (Delgado et al., 2001), or septic shock. This neuropeptide belongs to the structural-related peptide named secretin/VIP family (Table 2) encompassing VIP, pituitary adenylate cyclase activating peptide (PACAP), secretin, growth hormone releasing factor (GRF), peptide having an histidine residue in N-terminal position and an isoleucine residue in C-terminal position (PHI and its human homolog PHM), helodermin, glucagon, gastric inhibitory polypeptide (GIP), glucagon-like peptide 1 and 2 (GLP-1 and GLP-2).

**Table 1:** **Major physiological and pathophysiological actions of VIP^*a*^**.

Short-term	Neurotransmition, exocrine secretions (water, ions), hormone release (prolactin, luteinizing hormone, growth hormone, insulin…), muscle relaxation (vasodilator, bronchodilator, gastro–intestinal motility), metabolism
Long-term	Circadian rhythms, learning and behavior, growth regulator of whole fetuses and embryonic brain
Other effects	Neuroprotection, suppression of inflammation, immunomodulation, effects on cell proliferation in cancer

a Reviewed in Gozes et al. (2003); Dickson and Finlayson (2009); Delgado and Ganea (2011); Moody et al. (2011); Harmar et al. (2012).

**Table 2:**
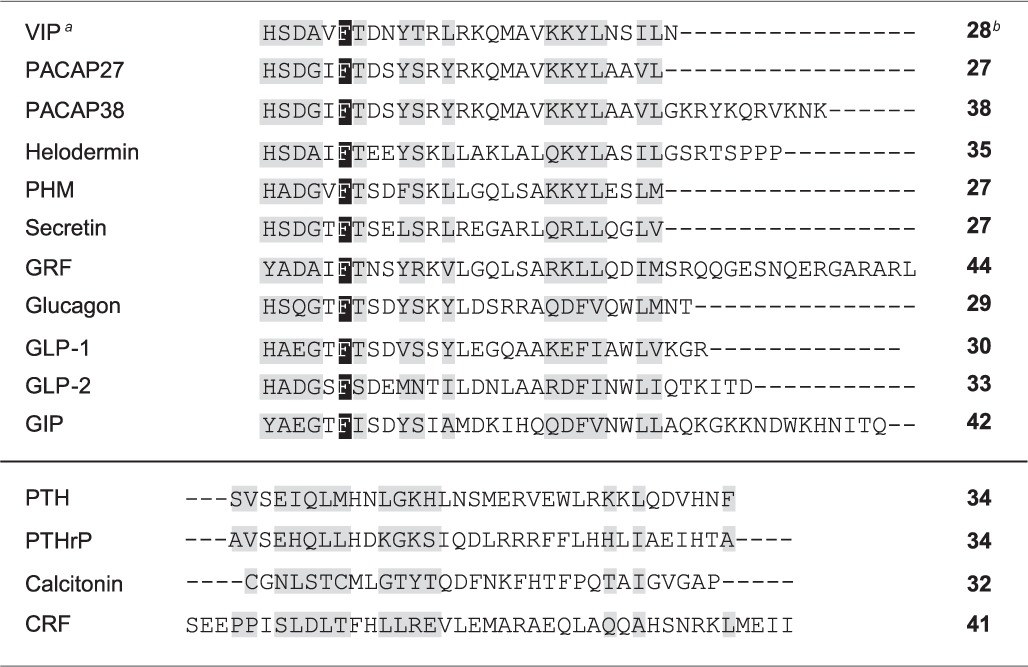
**Sequence alignments of class B GPCR ligands^*b*^**.

^a^Black boxes represent sequence identity and light gray boxes represent sequence homology.

^b^Numbers indicate the length of the peptides.

## VIP a potential therapeutical agents

Few years ago, VIP emerged as a potential therapeutic agent for various diseases including asthma (Groneberg et al., 2006), sexual impotence (Fahrenkrug, 2001), brain strokes (Dogrukol-Ak et al., 2004), chronic inflammation (Delgado et al., 2004), neurodegenerative disorders (Dejda et al., 2005), and cancers (Moody et al., 2011). Recently, a lot of reports have focused on the role of VIP and its receptors in chronic inflammation and neurodegenerative diseases.

VIP has been identified as a very promising agent in treatment of inflammatory diseases (Delgado et al., 2004). Indeed, VIP appeared to be a very potent anti-inflammatory peptide in animal models of various chronic inflammatory diseases (Couvineau and Laburthe, 2012a,b). The VIP anti-inflammatory effect has been widely studied (Delgado et al., 2004). These studies showed, in homeostasis condition, innate and adaptive immunity, that VIP can help to preserve the equilibrium between pro-inflammatory and anti-inflammatory response. In chronic inflammatory diseases (Crohn disease, rheumatoid polyarthritis, hepatitis, encephalomyelitis…) a modification of this equilibrium can be induce by various stimuli such as pathogenic agents, auto-immunity, environment, genetic background… which lead to the stimulation of production of pro-inflammatory cytokines (IL-17, IL-1, IFNγ, TNFα…) by macrophages and lymphocytes T (Th1 and Th17). Conversely, the anti-inflammatory response mediated by anti-inflammatory cytokines (IL-10, IL-4, IL-13, TGFβ…) secreted by lymphocytes T (Th2 and Treg), was strongly inhibited. The VIP anti-inflammatory effect involves a “rebalancing” of immune system (Firestein, 2001) by inhibition of pro-inflammatory response (Th1 and Th17) and stimulation of anti-inflammatory response mediated by Th2 and Tregs. In parallel, VIP induces an inhibitory effect on innate immunity by inhibition of production of pro-inflammatory cytokines and chemokines secreted by macrophage. Moreover, VIP is able to strongly inhibit the production of reactive oxygen species (ROS) induced by fMLP in monocytes (personnal data). Moreover, various reports clearly demonstrate that VIP promotes tolerance by inducing expansion of Treg cells (Leceta et al., 2007). Whereas, some reports reveal that VIP-deficient mice are resistant to the development of induced-encephalomyelitis or induced-endotoxemia indicating that in these conditions VIP plays unexpected permissive and/or pro-inflammatory actions (Abad et al., 2010, 2012). Despite this effect, VIP represents a potential anti-inflammatory agent that could be used in human therapeutic treatment, although the VIP anti-inflammatory effects have been mainly described in animal models (Couvineau and Laburthe, 2012a,b). Whereas, the major obstacle to the use of VIP in clinic therapies is its high sensitivity to protease degradation. Indeed, removing of the first residues by peptidases, such as dipeptidyl peptidase IV (DPPIV), induces a drastic loss of affinity of VIP peptide family (Lambeir et al., 2001). To circumvent these labile properties, VIP can be modified to increase its resistance to degradation by N-acylation of the peptide N-terminal end or by substitution of residues involved in proteolytic consensus sequences (dibasic doublets). Recent data indicate that PACAP N-terminal modifications confer resistance to DPPIV (Bourgault et al., 2008). In the same way acetylation of the VIP N-terminal end increase its stability in the presence of human serum (personal data). Other strategies consist to protect peptide against degradation by insertion of VIP into micelles or nanoparticles (Fernandez-Montesinos et al., 2009; Onyüksel et al., 2009). Despite these limitations, VIP has been tested in a phase I clinical trial for the treatment of acute respiratory distress syndrome and sepsis (id: NCT00004494, http://www.clinicaltrials.gov).

In the mid-1980s, the first report of VIP neuroprotection, demonstrated that this peptide is able to prevent neuronal death associated with electrical blockade induced by tetrodotoxin (TTX) addition to primary spinal cord cultures (Brenneman and Eiden, 1986). Further studies have demonstrated that VIP plays a neuroprotective effects in various neurodegenerative diseases developed in animal models including Alzheimer's disease (Gozes et al., 1996), Parkinson's disease or encephalomyelitis (Gonzalez-Rey et al., 2005; Tan and Waschek, 2011). Some of these VIP neuroprotective actions were associated with glial cells possessing VPAC receptors. Clearly, VIP induced, on glial cells, a secretion of various trophic molecules having neuroprotective properties such as IL-1, IL-6, protease nexin-1, the chemokine RANTES and MIP (Dejda et al., 2005). Moreover, VIP inhibits the production of pro-inflammatory cytokines as TNFα and/or IL-1β secreted by activated microglia which is involved in neuroinflammation observed in Parkinson's disease or brain trauma models (Delgado et al., 2004). VIP also induces neuroprotective effect by increasing the secretion of activity-dependent neurotrophic factor (ADNF) and/or activity-dependent neurotrophic protein (ADNP) (Gozes et al., 2003). These two protective proteins, which belong to the heat shock protein family, are able to prevent the neuronal death (Dejda et al., 2005) and represent one of the most potent neuroprotective agents secreted by astroglia in response to VIP. Recently, it was suggested that the VPAC2 receptor, which binds VIP and/or PACAP with the same affinity, could be a potential target for the development of anti-psychotic drugs. Effectively, the VPAC2 receptor gene has been found to be duplicated in schizophrenia (Vacic et al., 2011). Although VIP is able to cross the brain blood barrier (Dogrukol-Ak et al., 2004), no clinical trials in humans were developed to evaluate its neuroprotective role in brain diseases. However, some human clinical trials based on VIP vasoactive properties on cerebral arteries and hemodynamics have been performed (id: NCT00272896 and NCT00255320 http://www.clinicaltrials.gov) to evaluate its role in development of headache/migraine.

## VPAC receptors, a representative members of class B GPCR

Biological responses induced by VIP are triggered by interaction with two receptors, VPAC1 and VPAC2, which are mainly coupled to the G-protein, Gs, resulting in the stimulation of cell adenylyl cyclase (Couvineau et al., 2010). Furthermore, some groups have reported the ability of VIP to increase calcium levels in different cells (Dickson and Finlayson, 2009). Moreover, VPAC1 receptor is able to interact with RAMP (Receptor Activity-Modifying Proteins) proteins, in particular RAMP2, inducing a significant enhancement of agonist-mediated inositol triphosphate production but do not modify the coupling to adenylate cyclase (Christopoulos et al., 2002). VPAC1 and VPAC2 receptors bind, with the same affinity, VIP and another neuropeptide named PACAP. It should be noted that VIP interacts also with the specific PACAP receptor (PAC1) but with a lower affinity (Couvineau and Laburthe, 2012a). Previous report indicate that VPAC1 receptor is able to homo-dimerize and hetero-dimerize with VPAC2 or secretin receptors (Harikumar et al., 2006). However, the relation between receptor oligomerization and the ability to VPAC1 receptor to interact with RAMPs remains unclear.

In the nineties secretin and VPAC receptors have been cloned (Ishihara et al., 1991, 1992; Lutz et al., 1993; Sreedharan et al., 1993; Couvineau et al., 1994) revealing a new G protein-coupled receptor (GPCR) sub-family termed class B GPCR. This GPCR sub-family shares with the other GPCR classes (A, C, D, E, F) the same general structural scheme characterized by the presence of seven-transmembrane helices denoted as TM I through TM VII which are interconnected by extracellular and intracellular loops (Fredrikson and Schiöth, 2006). The class B receptors family is composed of 15 members including receptors for VIP, PACAP, secretin, glucagon, glucagon-like peptide-1, glucagon-like peptide-2, GRF, GIP, and also include receptors for parathyroid hormone, calcitonin, calcitonin gene-related peptide, and corticotropin-releasing factor (CRF) (Couvineau and Laburthe, 2012b). Class B receptors display very low sequence homologies with others GPCRs (Laburthe et al., 2007) and share several specific characteristics: the presence of a large (>120 residues) and structured N-terminal ectodomain (N-ted) which is usually small in most class A GPCRs. The N-teds contain six highly conserved cysteine residues connected by three disulfide bridges, this sequence is the signature of class B GPCRs. The N-ted of class B receptor which represents the major binding site for its cognate natural peptide ligand, is characterized by; (1) the presence of a signal peptide probably involved in insertion of receptor in plasma membrane; (2) the absence of archetypical class A GPCR motifs such as E/D-R-Y or NP-xx(x)-Y; (3) a complex gene organization with many introns (Laburthe et al., 2002).

**Figure 1:**
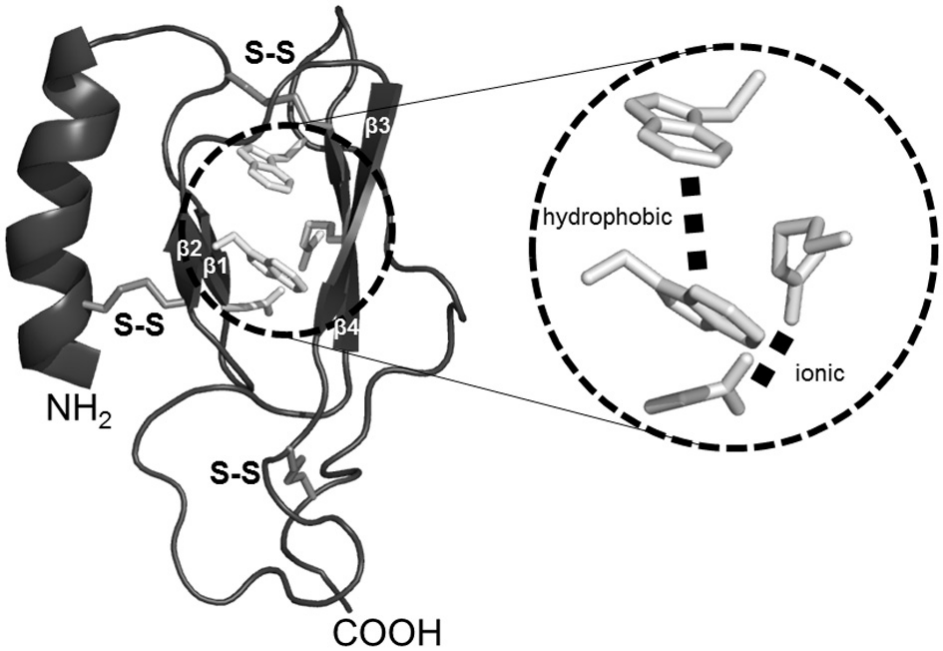
**Representation of generic Sushi domain core of Class B GPCR N-ted**. Common structural elements of class B GPCR N-ted are represented by the presence of (1) a N-terminal α-helix (*black* ribbon); (2) two anti-parallel β-sheets (β1-β2 and β3-β4, *black* ribbon). The Sushi domain structural core is stabilized by the presence of three conserved disulfide bonds (*middle gray* sticks) and represented in magnified inset an ionic and hydrophobic interactions (*light gray* sticks). All figures were obtained by using PyMOL software (http://www.pymol.org).

**Figure 2:**
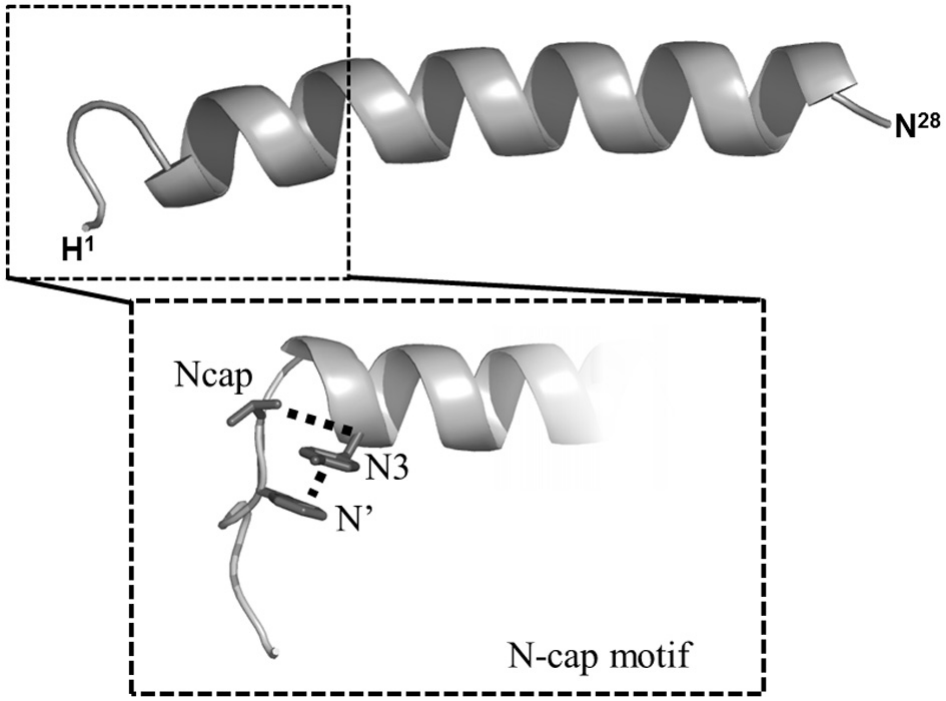
**NMR structure of VIP and representation of N-capping motif**. *Middle gray* ribbon represents the average conformation of VIP structure. In magnified inset the N-capping motif is represented as (1) the hydrophobic interactions between side-chain groups of N' and N3 residues (dashed lines); (2) the hydrogen bond between side chain of N-cap residue and backbone atom of N3 residue. See ref. Neumann et al. (2008) for details.

Currently, no data are available regarding the full-length structure of class B receptors as compare to class A receptors (Shoichet and Kobilka, 2012), although the structural properties of the class B GPCR N-ted, have recently been described, representing the first step toward better understanding of the binding receptor site at the atomic level. Recently, six N-ted structures, including those of the human PACAP receptor (PAC1), human PTH receptor (PTH1R), human GLP-1 receptor (GLP-1R), human GIP receptor (GIPR), and human type-1 and type-2 CRF receptor (CRFR1 and CRFR2) have been elucidated by Nuclear Magnetic Resonance (NMR) spectroscopy and X-ray crystallography in the presence of bound antagonist or agonists (Couvineau et al., 2010). These studies reveal the presence in the N-ted of a common core (Figure 1) formed by a Sushi domain (Parthier et al., 2009; Pal et al., 2012). This shared structure is composed of two anti-parallel β sheets (Figure 1) stabilized by (1) three disulfide bonds involving the typical six highly conserved cysteine residues (Figure 1); (2) a putative salt bridge involving acidic and basic residues, sandwiched between hydrophobic aromatic rings (Figure 1). The high conservation of the Sushi domain in the N-ted of class B GPCRs supports the idea that this structure plays a crucial role for peptide recognition (Grace et al., 2004). A “two-site” model for the binding of native ligands to class B GPCRs has been postulated (Hoare, 2005). In short, the central and the C-terminal portions of the peptide ligand are captured by the N-ted of the class B GPCRs. This step is essential for the peptide structuration, allowing the ligand N-terminus to interact with the transmembrane region of the receptor (Hoare, 2005).

As mentioned above VIP belongs to the secretin/VIP/PACAP family. The emergence of the class B GPCR has enlarged this peptide family (Table 2) by including parathyroid hormone (PTH), calcitonin, and CRF. All these natural ligands share some common properties: (1) they are all peptides with 27–44 amino acid residues; (2) they are synthesized and released by endocrine cells, neurons, and/or immune cells; (3) all these peptides exhibit a marked propensity to form α-helices; (4) all these peptides contain a N-Cap structure in the N-terminal part (Neumann et al., 2008). The presence of this structural signature which includes a hydrophobic cluster between N-terminal hydrophobic residues and a hydrogen bond between two polar residues (Figure 2) have been recently confirmed (Watkins et al., 2012). All these peptides play an important role in physiological processes and strongly impact human physiopathology including chronic inflammation diseases, neurodegenerative disorders, schizophrenia, diabetes, osteoporosis, stress (Couvineau and Laburthe, 2012a).

Cloning of the human VPAC1 receptor (Couvineau et al., 1994) allowed its extensive studied for many years by site-directed mutagenesis and molecular chimerism (Laburthe et al., 2007) laying its molecular basis in terms of: (1) affinity (Couvineau et al., 1995); (2) specificity (Couvineau et al., 1996a); (3) cellular addressing (Couvineau et al., 2004); (4) desensitization (Marie et al., 2003); (5) association with RAMP proteins (Christopoulos et al., 2002); (6) adenylyl cyclase coupling (Couvineau et al., 2003). These studies have revealed that the receptor N-ted plays a crucial role in peptide agonist binding (Couvineau et al., 2010). In parallel, structure–function relationships analysis of VIP by a complete alanine scanning (Nicole et al., 2000) showed that the peptide has a diffuse pharmacophoric domain. In this study we have demonstrated that the N-terminal 1–5 segment plays a crucial role in receptor activation e.g., mainly adenylyl cyclase activation.

## The VPAC1 binding site, contribution of photolabeling approach

**Figure 3:**
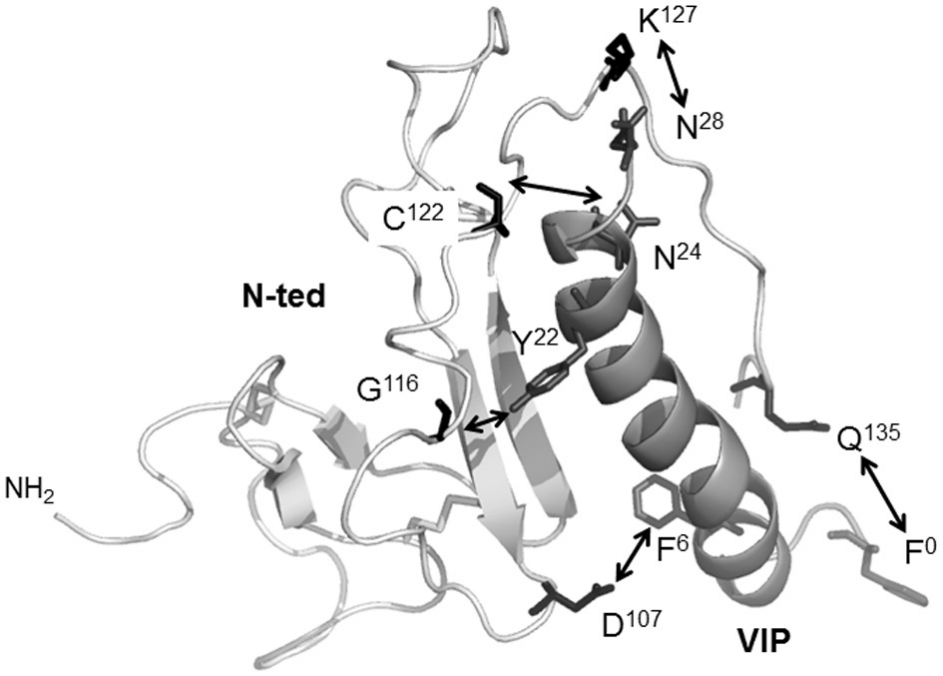
**The 3D-structural model of VPAC1 receptor N-ted and docking of VIP**. Ribbon representation of the VPAC1 N-ted: *light gray ribbon*, main chain; *white ribbon*, VIP. Docking calculations showed that Q^135^, D^107^, G^116^, C^122^, and K^127^ residues (*middle gray sticks*) present in the N-ted were in contact (*white arrows*) with the side chains of F^0^, F^6^, Y^22^, N^24^, and N^28^ (*black sticks)* of VIP residues, respectively. Figure was obtained by using PyMOL software (http://www.pymol.org).

The physical interaction sites between VIP and the VPAC1 receptor had remained elusive until the development of a photoaffinity labeling strategy, which allowed the demonstration that VIP side chains are physically in contact with the N-ted of VPAC1 (Couvineau et al., 2010). This strategy has two advantages over structural studies of purified recombinant receptors or receptor fragments: (1) the labeled ligand has an affinity for its receptor in the nanomolar range, which is similar to the high affinity measured under physiological conditions; and (2) the labeled ligand can interact with the glycosylated native receptor expressed in plasma membranes of eukaryotic cells. This is particularly important, given to the critical role of glycosylation in VPAC1 expression and function (Couvineau et al., 1996b). Addition of a benzophenone group (Bpa) to the VIP peptide has extensively contributed to the elucidation VIP biochemistry and of its receptor (Couvineau and Laburthe, 2012b). The use of photolabeling probes has clearly demonstrated that VIP residues in position 0, 6, 22, 24, or 28 were in physical contact with different amino acids in N-ted of the VPAC1 e.g., Gln^135^, Asp^107^, Gly^116^, Cys^122^, and Lys^127^ (Figure 3), respectively (Tan et al., 2003, 2004, 2006; Ceraudo et al., 2008, 2012). To dock VIP within the receptor N-ted, we determined the structure of VIP by NMR (Figure 2) and also developed a structural model of the VPAC1 receptor N-ted (Tan et al., 2006). Determination of VIP structure by NMR revealed that most of the 28 amino acids sequence has an α-helice structure (sequence 7–28) with the exception of the N-terminal 1–5 sequence, which has no defined structure in solution (Figure 2). In parallel, the structural model development of the VPAC1 receptor N-ted, by homology with the NMR structure of the CRF 2β receptor N-ted, allowed us to localize the VIP binding site in the N-ted. As expected, the structure contains two anti-parallel β sheets that are stabilized by three disulfide bonds between residues Cys^50^ and Cys^72^, Cys^63^ and Cys^105^, and Cys^86^ and Cys^122^, and by a putative salt bridge involving Asp^68^-Arg^103^, sandwiched between the aromatic rings of Trp^73^ and Trp^110^ (Figure 3). The NMR structure of VIP has been docked in the VPAC1 receptor N-ted giving rise to a valid model in which, the N-ted C-terminal part, nicely accommodates the VIP molecule at least for the 6-28 sequence (Figure 3). This model has been submitted to molecular dynamic simulations over 14 ns in a water box and appears to be highly stable (Ceraudo et al., 2008).

Recently, using similar strategy, we have characterized the interaction site of the VPAC1 receptor-specific VIP antagonist, [Ac-His^1^, D-Phe^2^, K^15^, R^16^, L^27^] VIP(3-7)/GRF(8-27) or PG 97-269 (Gourlet et al., 1997). This antagonist is a chimeric peptide between VIP (sequence 1–7) and GRF (sequence 8–27) having a D-phenylalanine residue in position 2. The use of Bpa^0^-PG97-269 affinity labeling probe revealed that the N-terminal part of antagonist physically interacted with Gly^62^ residue of VPAC1 N-ted (Ceraudo et al., 2012). These observations clearly support that the N-terminal part of VIP (agonist) or PG97-269 (antagonist) were recognized by two different domains present in N-ted of VPAC1 receptor.

As mentioned above, the N-ted structure of different class B GPCRs has been obtained recently by X-ray crystallography or NMR spectroscopy (Parthier et al., 2009). These studies seem to indicate the existence of two different binding sites for ligands in class B receptor N-teds (Couvineau et al., 2010). Analysis of these structure and/or molecular models revealed that N-teds of GIPR, PTHR, CRF1R, CRF2R, and GLP-1R interact with ligands in regions encompassing the loop located between β1 and β2 sheets and the loop located between β3 and β4 sheets (Parthier et al., 2009). In contrast, the N-teds of PAC1R and VPAC1R bind peptides along β3 and β4 sheets of the sushi domain (Couvineau et al., 2010). However, a recent report based on the X-ray crystallography analysis of PAC1 receptor N-ted and the docking of PACAP indicates that PACAP could interact with its receptor as GIPR, PTHR, CRF1R, CRF2R, and GLP-1R (Kumar et al., 2011). The real significance of these differences were unclear but may be tentatively related to the following interpretations: (1) some structural determinations were carried-out in presence of ligands which have a low affinity (micromolar range) for the recombinant N-ted whereas in other studies ligand affinity was higher; it also could be hypothesized that low and high affinity binding occur at different sites in the N-ted structure; (2) the determination of interaction between N-teds and ligands was mainly obtained in the presence of antagonist but it some cases in the presence of an agonist; (3) moreover it could be hypothesized that agonists and antagonists bind to different domains in the N-teds. Finally, we cannot exclude the possibility that ligands can bind by two different ways to N-ted of class B GPCR.

## The key role of the first transmembrane domain of VPAC1 in VIP binding

**Figure 4:**
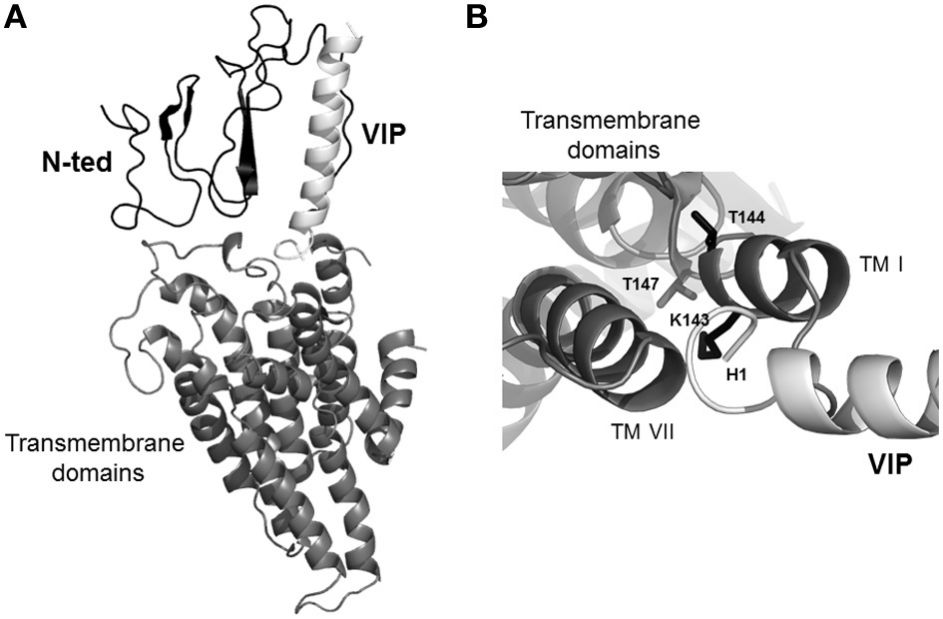
**Representation of global 3D-model of VPAC1 docked to VIP**. **(A)** 3D-global model of human VPAC1 based on X-ray structure of the A2A receptor. **(B)** Upside view of 3D-global model of human VPAC1 displaying side-chains of residues of the 3D-global model of the receptor (*black sticks*) in close contact (distance <6Å) with the N-terminal end of VIP (*white*). *middle gray ribbon*, transmembrane domains; *black ribbon*, main chain of N-ted; *white ribbon*, main chain of VIP.

VPAC1 domain interacting with the N-terminus of VIP (1–5) is still unknown. Up to now, no data are available regarding the full-length structure of class B receptors. To circumvent this unavailability, a 3D-model of the receptor encompassing VIP/N-ted complex and the transmembrane core of the receptor (Figure 4) was developed (Ceraudo et al., 2012). The 3D-model of the transmembrane core was constructed by homology modeling based on the recent determination of the X-ray structure of the adenosine A2A receptor (Jaakola et al., 2008). The resulting 3D-model of VPAC1 revealed that the central and C-terminal residues of VIP are in contact with N-ted whereas the N-terminus of VIP lies in a pocket formed by the extracellular side of the first, second and seventh transmembrane domains and the second extracellular loop of VPAC1 (Figure 4A). Based on distance calculation (<6Å) between residues of VPAC1 and VIP, substitutions by alanine of residues revealed that many residues are involved in the binding affinity of VIP to VPAC1. Three of them (H^112^, L^131^, and Q^134^) are present in the N-ted, and their substitution to alanine induced an affinity modification of about 100 times as compared to native receptor, indicating that these residues are probably involved in the interaction between the N-ted and the central and C-terminal parts of VIP (Ceraudo et al., 2012). Substitution to alanine of four other residues (K^143^, T^144^, T^147^, and L^375^) located in the extracellular side of TMI and VII, also induced a strong modification of receptor affinity for VIP (Ceraudo et al., 2012). Moreover site-directed mutagenesis experiments and reciprocal exchange between K^143^, T^144^, and T^147^ residues of VPAC1 and H^1^ of VIP, shown that this interaction with (Figure 4B) the first histidine residue of VIP play a crucial role (Ceraudo et al., 2012). This step is important for the adenylyl cyclase activation (Couvineau et al., 1984). These observations were in good agreement with previous results indicating that D^196^ present in the second extracellular loop (Du et al., 1997), K^195^ and R^188^ in TMII (Solano et al., 2001), N^229^ in TMIII and Q^380^ in TMVII of VPAC1 play an important role in VIP binding and probably could interact with the D^3^ residue of VIP (Chugunov et al., 2010). Thus, these results along with our data clearly indicate that the N-terminus of VIP interacts with the extracellular side of the VPAC1 core.

## The N-ted determines the specificity of the VPAC1 receptor

As mentioned above, VPAC1 and VPAC2 receptors do not discriminate between the two neuropeptides, VIP and PACAP. Moreover, some others VIP related-peptides are able to bind to human VPAC1 receptor with low affinity, including peptide histidine methionineamide (PHM), secretin, helodermin and GRF (Laburthe et al., 2007). Potency order being VIP = PACAP>helodermin>PHM>GRF>>secretin. In this context, the development of specific ligands for VPAC1 and VPAC2 receptors represents a major goal. To develop a specific VPAC1 agonist, structure-function relationships analysis of VIP by a complete alanine scanning (Nicole et al., 2000) was used to rationally design the most potent and specific peptide for VPAC1 receptor currently available e.g., [Ala^11,22,28^]-VIP (Nicole et al., 2000). Indeed, this VIP derivative has an affinity 1000 times higher for the VPAC1 receptor, which is mainly involved in VIP anti-inflammatory action, than VPAC2 receptor (Delgado et al., 2004). As mentioned above a high selective antagonist of VPAC1 receptor (PG97-269) has been developed (Gourlet et al., 1997). Regarding VPAC2 receptor, the cyclic peptide analog of VIP [Ac-Glu^8^, OCH3-Tyr^10^, Lys^12^, Nle^17^, Ala^19^, Asp^25^, Leu^26^, -Lys^27,28^ -VIP(cyclo 21–25)] or Ro 25–1392 is a potent and selective agonist (Xia et al., 1997). In our opinion, there is still no satisfactory VPAC2 receptor antagonist since PG 99–465, a VIP analog that antagonizes VIP action on VPAC2 receptor, which also has a significant agonist activity on human VPAC1 receptor (Moreno et al., 2000). Since recently, two non-peptide antagonists specific of VPAC1 (Harikrishnan et al., 2012) or VPAC2 (Chu et al., 2010) have been developed but they display a very low affinity for receptors.

The use of VIP photoaffinity probes associated to receptor mapping and Edman degradation demonstrated that VIP physically interacts with the N-ted of VPAC1 receptor (Couvineau et al., 2010). In order to get a high resolution structure of the VPAC1 receptor N-ted, the production of large quantities of recombinant N-ted protein in bacteria was performed (Couvineau et al., 2008). The 31–144 sequence of human VPAC1 receptor corresponding to the N-ted sequence in which the signal peptide (Couvineau et al., 2004) has been deleted was subcloned in front of 6xHis (His-tag) and behind the thioredoxin sequence containing a thrombin cleavage site (Couvineau et al., 2008). The construction of the thioredoxin-N-ted-6xHis (Trx-N-ted-6xHis) fusion protein was chosen in order to increase the solubility of recombinant proteins as previously described for production of recombinant N-ted of mouse CRF2β receptor (Grace et al., 2004). The soluble recombinant N-ted was purified onto Ni-NTA column and tested for its ability to bind VIP by using the influence of VIP binding on the intrinsic tryptophan fluorescence (ITF) of W^67^, W^73^, and W^110^ residues which are present in the N-ted sequence. Indeed, the presence of three tryptophan residues in the N-ted Sushi domain (Couvineau et al., 2008) represents a good fluorescent tag which can be used to measure the interaction between VIP and recombinant N-ted. Based to the ITF parameters, the estimation of dissociation constants revealed a Kd of 0.54 μM for VIP, 0.57 μM for PACAP, and 1 μM for PG96-269 (Couvineau et al., 2008). It should be noted that those Kd values were close to Kd values observed for others purified N-ted such as PAC1 receptor (Sun et al., 2007) and GIP receptor (Parthier et al., 2007). Moreover, the Kd of truncated VIP6–28 is very similar to Kd of native VIP (0.54 μM vs. 0.85 μM) demonstrating that the 6–28 VIP sequence is sufficient to interact with a low affinity to recombinant N-ted (Table 3). In contrast, the deletion of large C-terminal (VIP1-12), central (VIP1-9/21-28) and N-terminal (VIP18-28) part of VIP abolishes totally the ability of truncated peptides to bind to recombinant N-ted (Table 3). These data clearly indicate that recombinant N-ted is able to recognize with a low affinity the central and C-terminal part of VIP molecule. Using the same approach, the ability of VPAC1 recombinant N-ted to discriminate VIP related-peptides was investigated (Table 3). As shown in Table 2, the estimation of Kd was of 0.54 μM, 0.57 μM, 2.54 μM, 8 μM, 10.12 μM, and 16 μM for VIP, PACAP, helodermin, PHM, GRF, and secretin respectively, indicating that the order of potency is similar to native receptor i.e., VIP = PACAP>helodermin>PHM>GRF>secretin (Laburthe et al., 2007). Taken altogether these results reveal that: (1) the recombinant N-ted is able to bind with a low affinity and to discriminate VIP related-peptides suggesting that the VPAC1 N-ted contains residues involved in the VPAC1 specificity; (2) the first transmembrane domain of VPAC1 contains three residues (see above) which interact with the first residue of VIP and these three residues are probably involved in the high affinity and the activation of the receptor (Figure 4B).

**Table 3:** **Binding of VIP related-peptides to recombinant N-ted**.

**Peptides**	**Kd (μM)^*a*^**
VIP	0.54 ± 0.09
PG97–269	1.05 ± 0.50
PACAP27	0.57 ± 0.06
Helodermine	2.54 ± 0.71
PHM	8.00 ± 2.00
GRF	10.12 ± 0.98
Secretin	16.00 ± 1.00
VIP2–28	0.85 ± 0.36
VIP1–12	ND^b^
VIP18–28	ND
VIP1–9/21–28	ND

a The ITF of W^67^, W^73^, and W^110^ residues from the purified N-ted was measured in 2 ml of HEPES buffer pH 7.5 containing 1 μM purified N-ted, in absence or presence of increasing concentration of peptides. Dissociation constants were determined from titration curves using analytical procedure developed by Bechet et al. (Bechet et al., 1986).

b Not detectable.

## Conclusion

The VPAC receptors, in particular VPAC1, are very promising targets for the development of therapeutic molecules in various pathologies including asthma, chronic inflammation diseases (Crohn's disease, rhumatoid arthritis, septic shock, multiple sclerosis…) neurodegenerative disorders, schizophrenia. While new peptide derivatives specifically targeting VPAC receptor sub-types are now available, however, their very short half-life and the inconvenient related to their administration routes make them difficult to use in human therapy. The recent advance in the structural knowledge of the VPAC1 binding site should lead to the design of non-peptide receptor agonists and/or antagonists. The development of such molecules will represent an important overhang in the treatment of many human diseases.

### Conflict of interest statement

The authors declare that the research was conducted in the absence of any commercial or financial relationships that could be construed as a potential conflict of interest.

## List of non-standard abbreviations

VIP: Vasoactive Intestinal Peptide
PACAP: Pituitary Adenylate Cyclase Activating Peptide
VPAC: VIP and PACAP receptor
Bpa: Benzophenone
N-ted: N-terminal ectodomain
ITF: Intrinsic Tryptophan Fluorescence.
